# Oral Wound Healing Potential of *Polygoni Cuspidati Rhizoma et Radix* Decoction—In Vitro Study

**DOI:** 10.3390/ph16020267

**Published:** 2023-02-10

**Authors:** Jakub Hadzik, Anna Choromańska, Bożena Karolewicz, Adam Matkowski, Marzena Dominiak, Adrianna Złocińska, Izabela Nawrot-Hadzik

**Affiliations:** 1 Department of Dental Surgery, Wroclaw Medical University, 50-425 Wroclaw, Poland; 2 Department of Molecular and Cellular Biology, Faculty of Pharmacy, Wroclaw Medical University, 50-556 Wroclaw, Poland; 3 Department of Drug Form Technology, Wroclaw Medical University, 50-556 Wroclaw, Poland; 4 Department of Pharmaceutical Biology and Biotechnology, Faculty of Pharmacy, Wroclaw Medical University, 50-556 Wroclaw, Poland; 5 Laboratory of Elemental Analysis and Structural Research, Faculty of Pharmacy, Wroclaw Medical University, 50-556 Wroclaw, Poland

**Keywords:** *Reynoutria*, *Polygonum cuspidatum*, gingiva, wound healing, resveratrol, polysaccharides, oral wound, dentistry, medicinal plant, pharmacopoeial drug, traditional medicine

## Abstract

*Polygoni Cuspidati Rhizoma et Radix* (*syn.* rhizomes of *Reynoutria japonica* Houtt.) is a pharmacopoeial raw material in Europe and China. In traditional medicine, one of the applications for *Reynoutria japonica* rhizomes is wound healing. In a recent in vitro study, we demonstrated that ethanol and acetone extracts from this herbal drug have the potential to heal oral gum wounds. However, considering that a majority of herbal medicines have been traditionally administered as water decoctions, in the present study, a decoction of *Reynoutria japonica* rhizomes was prepared and detailed tests to determine its in vitro gingival wound healing activity were conducted. We used the primary human gingival fibroblasts (HGF) incubated with a decoction to determine cell viability (MTT assay), cell proliferation (the confocal laser scanning microscope—CLSM), and cell migration (wound healing assay). Moreover, the collagen type III expression was examined using immunocytochemical staining. The studied decoction was qualitatively and quantitatively characterized using the validated HPLC/DAD/ESI-HR-QTOF-MS method. The Folin–Ciocalteu assay was used to determine the total phenols and tannins content. Additionally, HPLC-RI analysis of decoction and the previously obtained ethanol and acetone extracts was used to determine the composition of saccharides. Low concentration (from 50 to 1000 µg/mL) of decoction after 24 h incubation caused a significant increase in HGF cell viability. No cytotoxic effect was observed at any tested concentration (up to 2000 µg/mL). The lowest active concentration of decoction (50 µg/mL) was selected for further experiments. It significantly stimulated human gingival fibroblasts to proliferate, migrate, and increase the synthesis of collagen III. Phytochemical analysis showed significantly fewer polyphenols in the decoction than in the ethanol and acetone extracts tested earlier. In contrast, high levels of polysaccharides were observed. In our opinion, they may have a significant effect on the oral wound healing parameters analyzed in vitro. The results obtained encourage the use of this raw material in its traditional, safe form—decoction.

## 1. Introduction

The rhizome with roots of *Reynoutria japonica* Houtt. is a traditional Chinese medicinal herb (*hu zhang* in pinyin Chinese) listed in the European Pharmacopoeia under the name *Polygoni cuspidati rhizoma et radix.* This herbal medicine was included in the European Pharmacopoeia in 2017. According to Pharmacopoeia, a minimum of 1.0% emodin content and 1.5% of polydatin content in the dried herb are required as a quality check [1]. Traditionally, hu zhang has been used in East Asia against many diseases, such as hyperlipemia, inflammation, infection, and for wound healing [2]. According to current knowledge, the most important bioactive compounds in *Polygoni cuspidati rhizoma et radix* are stilbenes (with a high concentration of resveratrol), anthraquinones, procyanidins, phenylpropanoid disaccharide esters, and other polyphenols [2,3,4,5]. To obtain a high content of these constituents, different percentages of ethanol/methanol or acetone are used in the extraction process [3,6].

In the previous in vitro study, our research team reported the gingival wound healing activity of 25% and 40% ethanolic as well as 60% acetone extracts from the *Reynoutria japonica* rhizomes [7]. The extracts stimulated human gingival fibroblasts (HGF) to proliferate and migrate, as well as increasing the synthesis of collagen III, but with different potency. The highest stimulation of proliferative and migratory activity was observed after incubation with 25% EtOH extract. This effect may have been related to the highest resveratrol content and the favorable composition of procyanidins. Currently, our attention has been drawn to the fact that although the overwhelming majority of Chinese herbal medicines (CHMs) are traditionally administered as water decoctions (*tangs*), relatively little research is done on them [8].

This experiment was designed to test whether a traditionally used *Polygoni cuspidati rhizoma et radix* decoction could have a stimulating effect on the oral wound healing process. The intraoral healing process is different from skin wound healing, so a different therapeutic approach is required. Intraoral wound healing aids are desirable for patients whose natural wound healing process is impaired, i.e., immunocompromised, post-transplant, post-radiotherapy or chemotherapy patients, and persons with systemic diseases [9,10]. Also, periodontal diseases, including periodontal and peri-implant disease [11], predispose to impaired wound healing due to high levels of oxidative stress, which impairs the proliferation and migration of HGFs [12,13]. Also, older age affects oral wound healing by impairing fibroblast proliferation, migration, and differentiation, and reducing collagen synthesis [14]. Moreover, prolonged wound healing in the oral cavity predisposes to bacterial invasion and wound infection, which can further inhibit the proper wound healing process. Due to the unsatisfactory results of currently available treatments, there is a need to search for new, effective therapies that promote intraoral healing [15,16].

In this study, we have prepared a decoction of *Reynoutria japonica* rhizomes according to the traditional recipe. We conducted a detailed study to determine its activity in healing gingival wounds in vitro using the same raw material and the same research methods as in our previous study [7]. Additionally, an HPLC-RI analysis of the decoction and the previously obtained extracts was used to determine the composition of saccharides.

## 2. Results and Discussion

### 2.1. Cell Viability—MTT Assay

**Figure 1:**
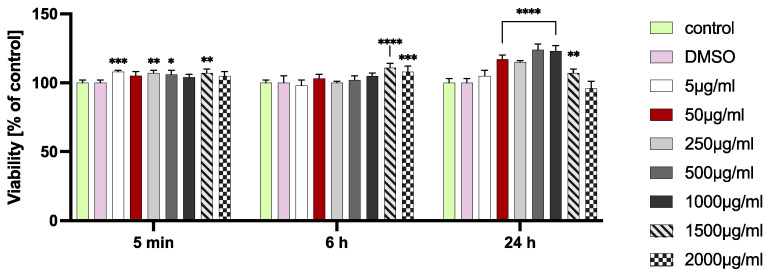
HGF viability after 5 min, 6 h, and 24 h incubation with the different concentrations of the decoction. Presented error bars are means ± SD for n ≥ 6. * Statistically significant compared to control at *p* ≤ 0.05, ** for *p* ≤ 0.01, *** for *p* ≤ 0.001, and **** for *p* ≤ 0.0001.

The *Reynoutria japonica* rhizome decoction was not cytotoxic to HGF cells at any tested concentrations and incubation time (Figure 1). It was different from the previously tested ethanol and acetone extracts, which at high concentrations—from 1000 µg/mL to 2000 µg/mL—showed a significant reduction in the viability of fibroblasts after 24 h of incubation [7]. The decoction at concentrations from 50 µg/mL to 1000 µg/mL, after 24 h of incubation, significantly increased the viability of HGF cells, up to 124% compared with that of the control. This stimulating effect on cellular metabolism has also been observed in previous studies with ethanol and acetone extracts from *R. japonica* rhizomes [5,7].

To check if fibroblasts proliferate after treatment with the decoction, we used a confocal laser scanning microscope to visualize changes in histone 3 expression. The lowest concentration (50 µg/mL) demonstrating a significant stimulation of the fibroblasts’ viability in the MTT test was selected for further studies (the same as in our previous research with solvent extracts [7]).

### 2.2. Confocal Laser Microscopy Study

**Figure 2:**
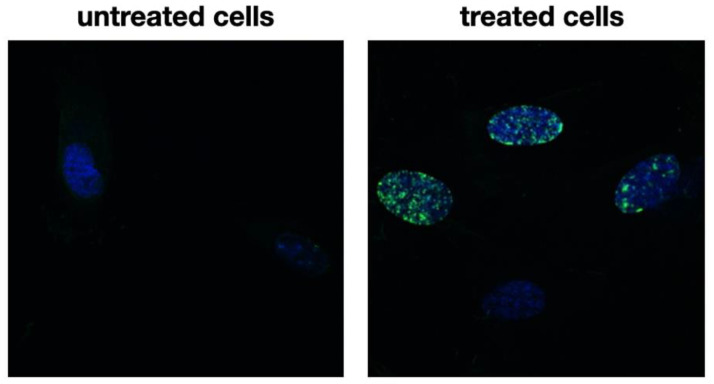
Microphotographs of HGF with histone H3 staining after 24 h of incubation with tested decoction (at a concentration of 50 µg/mL) and without decoction (untreated cells). Presented magnification ×600. Alexa Fluor 488 was used for histone-3 labeled, and DAPI was used for the cell nucleus labeled.

**Figure 3:**
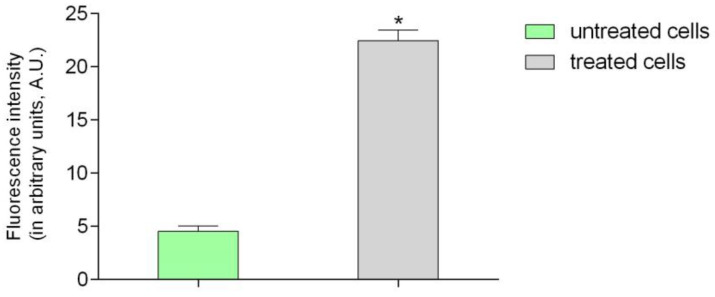
Fluorescence intensity (in arbitrary units) of histone H3 after 24 h incubation with tested decoction in fibroblast cells. Error bars shown in this figure are means ± SD for n ≥ 5. * Statistically significant at *p* < 0.0001 according to *t*-test.

Microphotographs of cells with immunofluorescent staining of histone H3 after 24 h incubation with tested decoction (at a concentration of 50 µg/mL) and without decoction (untreated cells) are presented in Figure 2. The primary rabbit polyclonal antibody anti-phospho-histone H3 was used to present an abundance of phosphorylation of serine 10 on histone (H3S10ph), the increase of which is observed during cell division [17,18]. Figure 3 shows the mean fluorescence intensity in arbitrary units for treated cells (22,488) and for untreated cells (4.515). The results indicate that the studied decoction significantly stimulated human gingival fibroblasts to divide. Importantly, the fluorescence intensity after incubation with the decoction was even higher than with 25% EtOH extract, which was the most active in our previous study [7].

### 2.3. Wound Healing Assay

**Figure 4:**
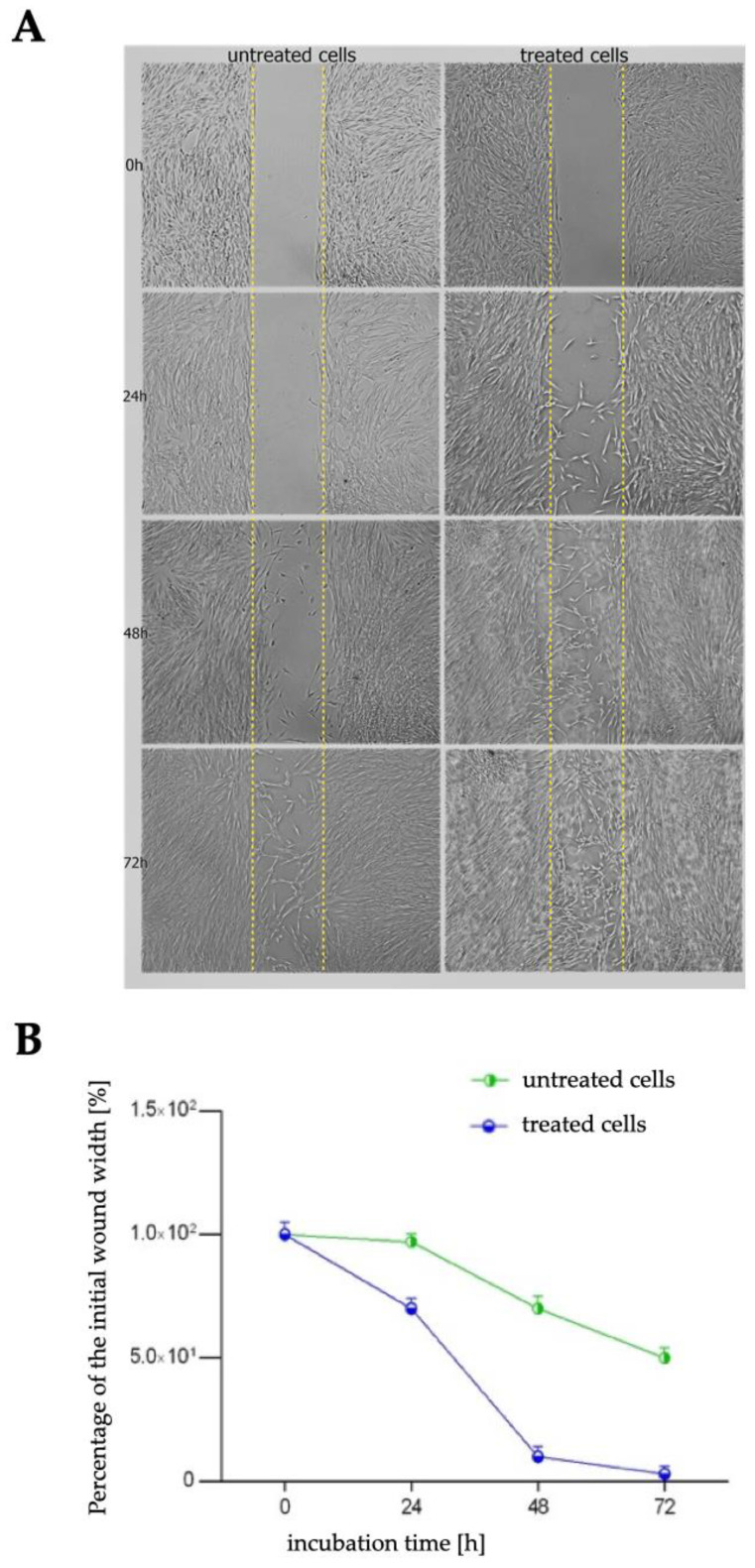
Effect of *R. japonica* rhizome decoction (at 50 µg/mL) on fibroblast motility investigated with wound healing assay in comparison to untreated cells. (**A**) Presentation of the migrating cells that invade the wound and (**B**) healed wound (%) as a function of time. ImageJ analysis software (NIH, Bethesda, MD, USA) was used. Data are mean ± SD (n = 3 replicates).

Proper proliferation and migration of gingival fibroblast cells play a significant role in the healing of oral cavity wounds. Using a cell migration-based wound healing assay, we analyzed the motility of the fibroblasts after incubation with 50 µg/mL of *R. japonica* decoction. Figure 4A shows the changes in the cell-covered area (gap closure) over time. Figure 4B illustrates the percentage of a healed wound as a function of time. Table 1 presents the percentage of wound (gap) closure over time.

### 2.4. Immunocytochemical Staining

**Figure 5:**
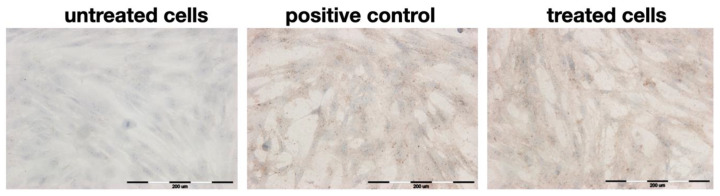
Microphotographs of HGF cells with immunocytochemically stained type III collagen after 24 h incubation with *R. japonica* decoction (at a concentration of 50 µg/mL). Positive control: betulinic acid (2 µM). Magnification on panel: ×200.

Gingival fibroblasts synthesize and secrete collagen type III, a high expression of which is observed during wound healing. Over time, type III collagen is reabsorbed and replaced by type I collagen, a key component of the extracellular matrix. Microphotographs (Figure 5) of HGF with immunocytochemically stained collagen type III show the influence of *R. japonica* decoction on expression of collagen type III. According to Table 2, which shows the semi-quantitative results, the 50 µg/mL of *R. japonica* decoction had a similar stimulating effect on collagen III production as 2 µM betulinic acid, used as the positive control.

In comparison to our previous study, the effect of *R. japonica* decoction on stimulation of fibroblasts to produce collagen III was similar to 60% acetone extract, slightly weaker than 40% EtOH, but stronger than 25% EtOH extract [7].

### 2.5. HPLC/DAD/ESI-HR-TOF-MS Analysis

**Figure 6:**
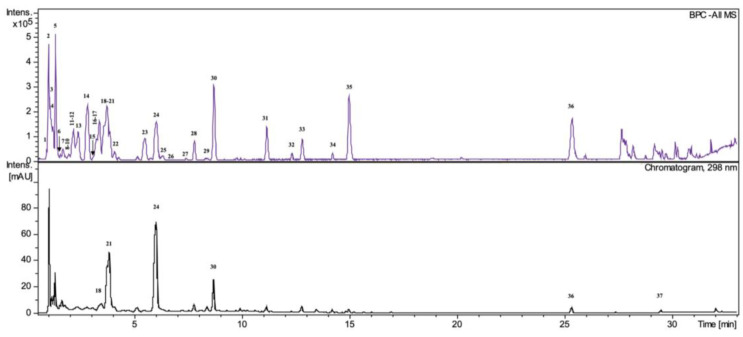
ESI-MS (negative mode) chromatograms (above) and UV-HPLC chromatograms (below) of decoction *R. japonica* rhizomes with detection at 298 nm. Key to peak identity as in Table 3.

To determine the composition of the compounds that contributed to the observed effects, qualitative and quantitative HPLC/DAD/ESI-HR-QTOF-MS analyses of decoction was performed. The UHPLC-QTOF-MS analysis revealed a total of 37 different compounds (Table 3, Figure 6) that belong to carbohydrates, stilbenes, flavan-3-ols, procyanidins, anthraquinones, organic acids, and naphthalenes.

Among all of the detected chromatographic peaks, seven remained unassigned and without a clear indication of their chemical nature. Two peaks were tentatively defined as carbohydrates. Most of the identified compounds were previously detected in extracts and fractions from rhizomes of *R. japonica*, as described in our previous reports [3,5,7,19]. The decoction was less diverse in terms of the content of compounds than the ethanol and acetone extracts analyzed in previous studies. It did not contain phenylpropanoids. Moreover, the trace amount of resveratrol was only noticed after extraction from the raw chromatogram. Stilbene glycosides and anthraquinone glycosides were present in greater amounts than their aglycones. In contrast, compound 31 with a high peak in the BPC (base peak chromatogram), labeled as unknown, with a deprotonated ion at *m*/*z* 253.0513 [M∓H]_−_ and predicted formula of C_15_H_9_O_4_ was not observed in the previously studied extracts. Product ions at *m*/*z* 225.0545 (C_14_H_9_O_3_, 0.3 ppm), *m*/*z* 224.0481 (C_14_H_8_O_3_, 1.26 ppm), *m*/*z* 209.0611 (C_14_H_9_O_2_, −1.2 ppm), *m*/*z* 197.0606 (C_13_H_9_O_2_, 0.9 ppm), *m*/*z* 169.0664 (C_12_H_9_O_,_ −3.0 ppm), and *m*/*z* 135.009 (C_7_H_3_O_3_, −1.5 ppm) suggest that it may be some kind of anthraquinone, i.e., rubiadin (HMDB0257354). In order to determine the structure of this compound, it must be isolated and subjected to detailed studies.

A previously developed, validated analytical method was used to quantify the selected compounds [19]. Piceid was detected in much lower amounts in the decoction than in previously tested ethanol or acetone extracts (Table 4). There was almost six times less piceid in the decoction than in the previously tested 25% EtOH extract and over ten times less than in the 60% acetone extract.

Resveratrol was not detectable without extraction from the chromatogram and was not quantifiable. Vanicoside A and B were not detected even in trace amounts Also, emodin and physcion were detected in much lower amounts than in ethanol and acetone extracts. Their content was sufficient for detection but not for correct quantification.

### 2.6. Total Polyphenols and Tannins Content

**Figure 7:**
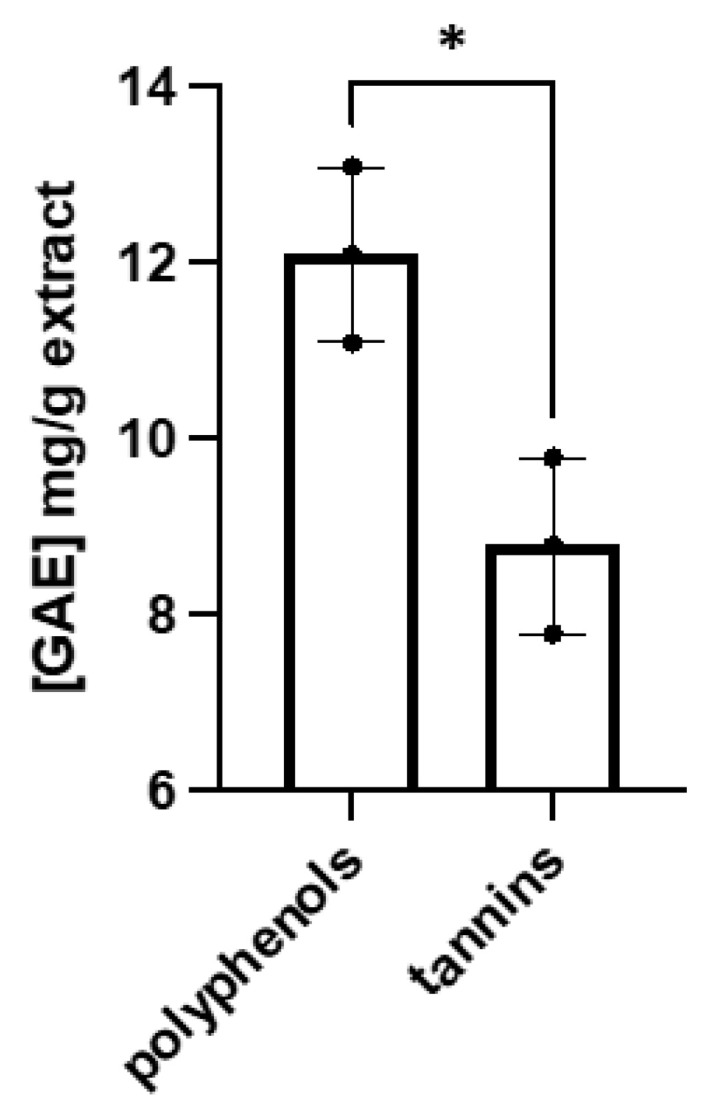
Total polyphenols and tannins content in studied decoction of *Reynoutria japonica* rhizomes. Error bars shown in this figure are means ± SD for n ≥ 3. * for statistically significant with *p* ≤ 0.05.

According to the modified Folin–Ciocalteu assay (Figure 7), total polyphenols and tannins make up a small part of the decoction.

### 2.7. HPLC-RI Analysis

**Figure 8:**
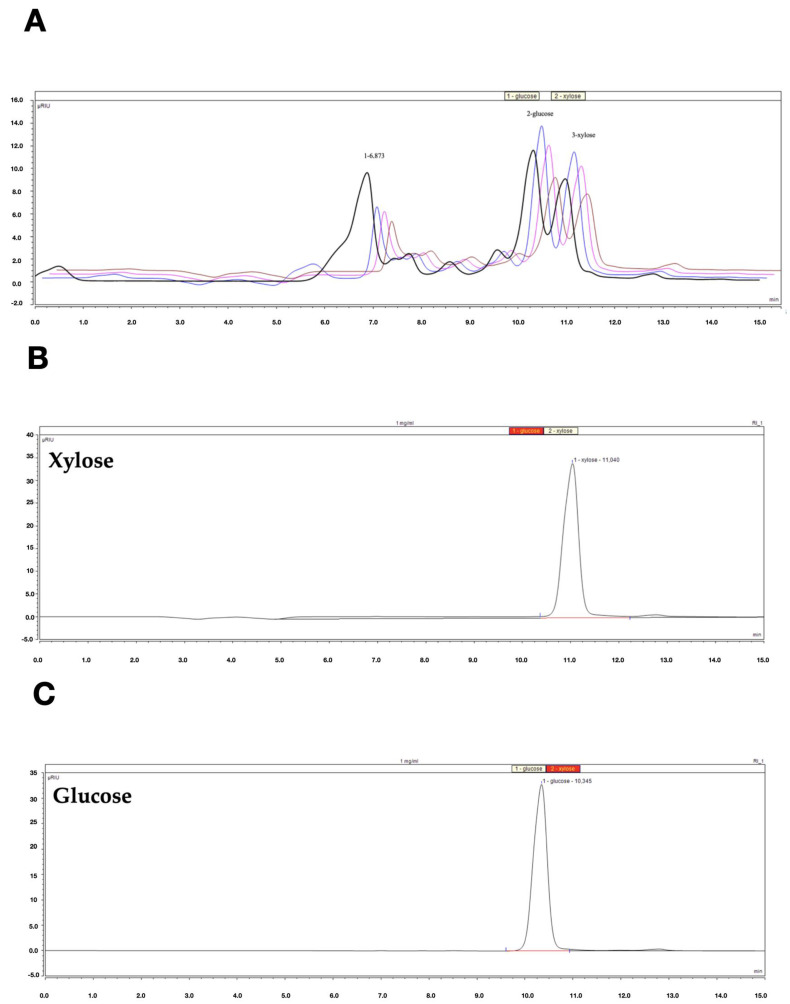

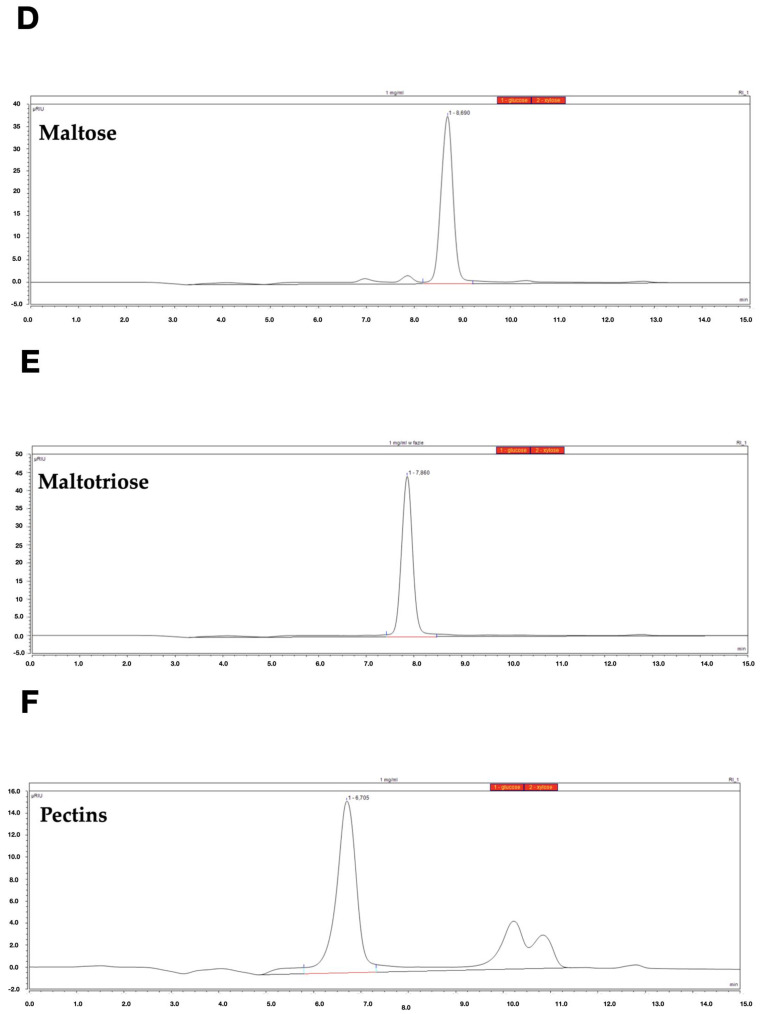
(**A**) HPLC-RI chromatograms of decoction (black) and previously studied extracts: 25% EtOH (blue), 40% EtOH (pink), 60% acetone (red). 1—unknown, 2—glucose, and 3—xylose. (**B**) HPLC-RI chromatogram of monosaccharide-xylose. (**C**) HPLC-RI chromatogram of monosaccharide-glucose. (**D**) HPLC-RI chromatogram of disaccharide-maltose. (**E**) HPLC-RI chromatogram of trisaccharide-maltotriose. (**F**) HPLC-RI chromatogram of polysaccharide-pectins.

The low content of polyphenols, including tannins, stilbenes, anthraquinones, phenylpropanoids, as well as the viscous consistency of the decoction encouraged us to examine its saccharide content. To compare the decoction with ethanol and acetone extracts from the previous study, the latter were also analyzed. The composition of saccharides in the studied samples was determined by high-pressure liquid chromatography with a refractometric detector (Figure 8A). All the extracts tested previously—25% EtOH (B), 40% EtOH (C), and 60% acetone (D) showed similar HPLC-RI chromatograms, which revealed the presence of three main compounds—glucose, xylose, and an unknown compound 1. The HPLC-RI chromatogram of the decoction differs from the extracts by the presence of a much larger unknown peak number 1 which also appears at a shorter retention time. To find out what kind of compound the unknown peak 1 is, multiple sugars were tested, among others: two monosaccharides (xylose (A) and glucose (B)), a disaccharide (maltose (C)), trisaccharide (maltotriose (D)), and polysaccharide (pectins (E)) (Figure 8B).

As shown above, under the chosen analysis conditions, the analytes flow from the Rezex ROA—Organic Acid H + column (Phenomenex) in the following order: first the most complex saccharides, such as the polysaccharide pectins, followed by trisaccharides, then disaccharides, and finally monosaccharides. We also confirmed this dependence on other compounds belonging to these sugar groups (data not presented). This is in accordance with the product specification of the Rezex ROA—Organic Acid H + column (Phenomenex). Our research shows that the decoction contained the greatest amount of polysaccharides. The exact identification of the polysaccharides and other saccharides present in the decoction requires further extensive research. At this point, however, it is important to consider whether they may have affected the activities under study.

The term polysaccharides covers a large group of compounds that are composed of monosaccharides (glucose, mannose, galactose, fructose, etc.). Depending on the type of monosaccharide, the polysaccharides can be divided into a homo-polysaccharide containing one type of monosaccharide and a hetero-polysaccharide containing two or more different types of monosaccharides. Monosaccharides are linked by glycosidic bonds to form long linear or branched structures. The literature can confirm the influence of polysaccharides on wound healing. There are reviews available [20,21] that show the skin wound healing activity of polysaccharides, most often obtained by hot water extraction, among others from such plants as *Trigonella foenum-graecum*, *Hammada scoparia*, *Linus usitatissimum*, *Avena sativa*, *Caesalpinia pulcherrima*, *Sanguisorba officinalis*, *Glycyrrhiza uralensis*, *Pimpinella anisum* as well as *Bletilla striata*, *Konjac*, *Eucommia ulmoides*, and *Mesona procumbens* [22]. Most of these studies have not established the exact chemical structure of the polysaccharides. Fewer data are available on the activity of polysaccharides in gingival wound healing. However, one of the polysaccharides with known gingival wound healing properties is acemannan from *Aloe vera*. Acemannan, a β-(1,4)-acetylated soluble polymannose has gingival wound healing properties proven in vitro and in vivo as well as in clinical studies [23]. A study by Jettanacheawchankit et al. [24] showed that acemannan induces proliferation and upregulation of growth factors (KGF-1, VEGF), and type I collagen expression in gingival fibroblasts as good reduction of wound area of experimental animals at day 7 after treatment. Another polysaccharide with a proven wound healing effect is β -glucan (glucose polymers linked by 1,3; 1,4 or 1,6 β-glycosidic bonds), which is a constituent of grains, yeast, and other fungi. In vitro studies have shown that β-glucans induced the proliferation and migration of keratinocytes and fibroblasts through specific receptors such as Dectin-1, CR3 or TLR. Preclinical animal studies have confirmed that it is effective as a wound healing agent [25].

The biological activity of the studied *Polygoni cuspidati rhizoma et radix* decoction was higher than that demonstrated by the ethanol and acetone extracts in our previous study, even though the decoction contained significantly fewer polyphenols [7]. On the other hand, the content of carbohydrates was significantly higher in the decoction than in either organic solvent extract. According to our best contemporary knowledge, there are no reports in the literature on polysaccharide contents in rhizomes of *R. japonica* and their biological activity. Notwithstanding, the methods used in this study were insufficient to determine the identity of the carbohydrates. Further research is therefore required to confirm our assumptions that polysaccharides in the decoction are largely responsible for stimulating gingival fibroblast cells to proliferate, migrate, and increase collagen III synthesis.

## 3. Materials and Methods

The present study was designed as an in vitro study on the human fibroblast cell line. The experimental protocol of a study “influence of extracts from medicinal plants *Reynoutria* on the functions of oral fibroblasts” was submitted to a Bioethics Commission of Wrocław Medical University (Wrocław, Poland) by a study principal investigator JH, and approved by the commission with the number KB-134/2020. The gingival biopsies for the cell study were provided by the Dental Clinical and Teaching facility of Dental Surgery Department at Wrocław Medical University. The study was conducted in full compliance with the GCP ICH: E6 (R2) and Declaration of Helsinki.

### 3.1. Plant Material and Decoction Preparation

*Reynoutria japonica* rhizomes harvested in Wroclaw (Poland) were used in the study. Details on the identification of the plant species, location, time of collection of raw material, and storage were presented in our previous study [7]. The same raw material was used in our previous study to prepare ethanol and acetone extracts [7]. Fifty grams of air-dried and powdered rhizomes of *R. japonica* were flooded with 500 mL of cold water and boiled under reflux for 20 min. After cooling down, the decoction was filtered through a paper filter and the solvent was evaporated under reduced pressure. An amount of 2.41 g of dry decoction was obtained. Stock solutions were prepared from the dried decoction by dissolving 100 mg of decoction in 1 mL of dimethyl sulfoxide (DMSO, Sigma-Aldrich, St. Louis, MO, USA). Different concentrations of the decoction (5–2000 µg/mL) were tested by taking the appropriate amount of the stock solution. As a solvent to prepare different concentrations of decoction, the cell culture medium was used.

### 3.2. Cell Culture

The study was carried out on the primary human gingival fibroblasts (HGF) obtained from the connective tissue of a dental patient’s hard palate. The details of the cell culture study were presented in our previous paper [7].

### 3.3. Cell Viability Assay

Fibroblast cell viability was assessed using the MTT (3-(4,5-dimethylthiazol-2-yl)-2,5-diphenyltetrazolium bromide) colorimetric assay (Sigma-Aldrich, Merck Group, Darmstadt, Germany). The assay was carried out as described in our previous work [7], except that a decoction was used instead of ethanol or acetone extracts. The decoction was used in the concentration range from 5 µg/mL to 2000 µg/mL. The concentration of DMSO in the samples was equal to or less than 2%, therefore, also 2% DMSO was used as a control in the study. For the experiment, the cells were seeded in 96-well microculture plates at 1 × 10^4^ cells/well. After the incubation with selected concentrations of decoction, the experiments were conducted according to the manufacturer’s protocol. The absorbance was determined at 570 nm.

### 3.4. Confocal Laser Microscopy Study

A confocal laser scanning microscope (CLSM, Olympus FluoView FV1000, Tokyo, Japan) was used to visualize histone 3 expression change after incubation with 50 µg/mL decoction. The study was performed exactly as described in our previous article [7], except that a decoction was used instead of acetone and ethanol extracts.

### 3.5. In Vitro Wound Healing Assay

Decoction at 50 µg/mL concentration was used for the wound healing assay as a fibroblast migration capacity test. The study was performed exactly as described in our previous article [7], except that a decoction was used instead of acetone and ethanol extracts.

### 3.6. Immunocytochemical Staining

Immunocytochemical staining was used to evaluate the expression of collagen type III after 24 h incubation with decoction at 50 µg/mL. Betulinic acid (2 µM) was used as a positive control. The study was performed exactly as described in our previous paper [7], except that a decoction was used instead of acetone and ethanol extracts. The intensity of immunohistochemical staining was evaluated as negative (−), weak (+), moderate (++), or strong (+++), as in the previous article [7,26].

### 3.7. HPLC/DAD/ESI-HR-QTOF-MS Analysis

The obtained decoction was prepared for the qualitative and quantitative analysis. An amount of 50 mg of dried decoction was dissolved in 80% methanol (MeOH, Merck / MilliporeSigma, Darmstadt, Germany) in a volumetric flask to obtain a 5 mg/mL concentration. After filtering the prepared solutions through a 0.22 μm syringe membrane (Chromafil, Macherey-Nagel, Düren, Germany) into vials, 4 μL of the sample was injected by autosampler into the high-pressure liquid chromatography (HPLC) system. The same HPLC-DAD-MS system was used as well as the same qualitative analysis conditions as in our previous study [7].

A previously developed, validated analytical method was used to quantify the selected compounds [19]. Linearity, the LOD (limit of detection), and LOQ (limit of quantification) for all quantified compounds were presented in our previous study [19].

### 3.8. Total Polyphenols and Tannins Content

The content of polyphenols and tannins was determined using the modified Folin–Ciocalteu assay based on Singleton and Rossi method [27]. The study was conducted in the same way as described in our previous articles [28,29].

### 3.9. HPLC-RI Analysis

The composition of saccharides in the studied samples was determined by high-pressure liquid chromatography (HPLC) according to the procedure described in the previous paper [30]. Briefly, the chromatographic method was performed using the Dionex Ultimate 3000 system (Thermo Fisher Scientific, Waltham, MA, USA). The content of saccharides was determined using a Rezex ROA—Organic Acid H+ column with the following dimensions: 300 mm × 7.8 mm i.d. and 8% cross-linked H + (Phenomenex, Torrance, CA, USA). The column was kept at 60 °C. The mobile phase was 5 mM sulfuric acid, previously filtered through a 0.45 mm membrane filter (Millipore). The flow rate was 0.6 mL/min. A refractometric detector ERC RefractoMax 520 (DataApex, Prague, Czech Republic) was used for analyte detection. All analytical determinations were performed in triplicate and mean values are given. An amount of 50 µL of decoction or extract (at 2.5 mg/mL concentration) dissolved in the mobile phase and filtered through a 0.22 µm Chromafil PTFE hydrophilic syringe membrane was injected into the HPLC system by an autosampler. Among others, the following compounds were analyzed as standards: glucose, xylose, maltose, maltotriose, and pectins (all from Merck/MilliporeSigma, Darmstadt, Germany). The standards were dissolved in the mobile phase in a volumetric flask to obtain a concentration of 1 mg/mL.

### 3.10. Statistical Analysis

All assays were performed in at least triplicate and results are presented as the mean of the replicates ± SD. The Shapiro–Wilk test was used to evaluate the distribution of results. Two-way ANOVA and Tukey’s multiple comparisons tests (GraphPad Prism v. 9, San Diego, CA, USA) were used to evaluate significant differences between the obtained values. In studies where only the means between the control and treatments were compared, the *t*-test was used.

## 4. Conclusions

The decoction of *Reynoutria japonica* rhizomes prepared according to a traditional recipe stimulated gingival fibroblasts to proliferate, migrate, and increase the synthesis of collagen III. Based on the phytochemical research (HPLC/DAD/ESI-HR-QTOF-MS, HPLC-RI analysis, and Folin–Ciocalteu assay), we can conclude that the high content of polysaccharides observed in the decoction may have an impact on their high wound-healing activity in vitro. Because this is the first published report on the presence of polysaccharides in this plant material, further research is needed to confirm our assumptions. Importantly, the decoction was found to be noncytotoxic to HGF cells at any tested concentrations and incubation time. The safety and high activity of the studied *Polygoni cuspidati rhizoma et radix* decoction encourage its future use in oral wound healing.

## Figures and Tables

**Table 1:** Wound (gap) closure percentage. Data obtained in ImageJ software.

	Time of the Incubation		
	24 h	48 h	72 h
untreated cells	3%	30%	50%
treated cells	30%	90%	97%

**Table 2:** The intensity of immunocytochemical reaction in human fibroblast cells using the antibodies against type III collagen after 24 h of exposure to the decoction (at a concentration of 50 µg/mL). Betulinic acid (2 µM) was used as positive control.

Sample	Intensity of Staining	HGF Stained [% ± SD]
untreated cells	−	96 ± 2
positive control	++	95 ± 1.5 *
decoction	++	99 ± 1 *

* *p* < 0.05 with reference to the control cells (Student’s *t*-test). The intensity of immunohistochemical staining was evaluated as negative (−) and moderate (++).

**Table 3:** The table shows the compounds identified in *Polygoni Cuspidati Rhizoma et Radix* decoction and their retention times, UV λ max, MS data, and ionic formulas.

Nr.	Compound	Rt. [min]	UV Max [nm]	*m*/*z* [M-H]^−^	Error (ppm)	Ion Formula	MS^2^ Main-Ion (Relative Intensity %)	MS^2^ Fragments (Relative Intensity %)	References
1	Unknown carbohydrate	1.0	ND	341.1093	−1.0	C_12_H_21_O_11_	113.0239(100)	119(80), 101(65), 179 (28), 173(21)	HMDB0000258
2	Unknown carbohydrate	1.05	ND	719.2028	1.8	C_30_H_39_O_20_	377.0864(100)	379(26), 341(13), 215 (1.9), 179(0.2)	-
3	Unknown	1.1	ND	439.0815	4.5	C_26_H_15_O_7_	96.9632(100)	241(2)	-
4	Organic acid e.g., malic acid	1.15	ND	133.0145	−2.2	C_4_H_5_O_5_	115.0058(100)		HMDB0000156
5	Organic acid e.g., citric acid	1.3	ND	191.0200	−1.4	C_6_H_7_O_7_	111.0109(100)		HMDB0000094
6	Unknown	1.57	225, 280	443.1931	−1.9	C_21_H_31_O_10_	189.0588(100)	119(7), 113(7), 101 (7)	-
7	Procyanidin dimer, Type B	1.6–1.8	225, 280	577.1358	−1.1	C_30_H_25_O_12_	289.0737(100)	407(60), 125(30), 425(18)	[3]
8	Procyanidin trimer, Type B	1.8–1.9	225, 280	865.197	1.8	C_45_H_37_O_18_	575(100)	287(92), 577(79), 695(57)	[3]
9	Procyanidin dimer, Type B	1.9–2.0	225, 280	577.1349	0.4	C_30_H_25_O_12_	289.0719(100)	407(68), 125(42), 425(20)	[3]
10	Procyanidin trimer, Type B	2.0–2.1	225, 280	865.1983	0.3	C_45_H_37_O_18_	577.1353(100)	287(90), 575(87), 865(75), 425(58), 289(45), 695(44), 713(42)	[3]
11	Catechin	2.1	225, 280	289.0721	−1.1	C_15_H_13_O_6_	123.0452(100)	109(92), 125(70), 245(35)	[3]
12	Procyanidin dimer, Type B	2.2	225, 280	577.1356	−0.8	C_30_H_25_O_12_	289.0744(100)	407(60), 125(34), 425(19)	[3]
13	Procyanidin dimer, Type B	2.4	225, 280	577.1363	−1.9	C_30_H_25_O_12_	289.0744(100)	407(61), 125(39), 425(16)	[3]
14	Epicatechin	2.8	225, 280	289.0723	−0.8	C_15_H_13_O_6_	109.0292(100)	123(93), 221(80), 125(76)	[3]
15	Procyanidin trimer, Type B	3.15	225, 280	865.1966	2.3	C_45_H_37_O_18_	577.1344(100)	287(85), 575(77), 865(64), 425(55), 713(44), 413(41), 695(40)	[3]
16	Unknown	3.25	280	499.1293	2.3	C_18_H_27_O_16_	499.1298(100)	97(10), 111(6)	-
17	Unknown	3.38	280	499.1291	2.6	C_18_H_27_O_16_	499.1299(100)	97(9), 111(5)	-
18	Piceatannol glucoside	3.4	220, 290, 318	405.1187	0.9	C_20_H_21_O_9_	243.0671(100)	244(15), 245(2), 201(1)	[19]
19	Unknown	3.6	282, 325	439.1085	1.8	C_16_H_23_O_14_	439.1089(100)	97(21), 424(2)	-
20	Procyanidin dimer monogallate	3.7	225, 280	729.1448	1.8	C_37_H_29_O_16_	407.0787(100)	289(46), 577(29), 451(21)	[3]
21	Resveratrolside	3.8	220, 304, 315	389.1237, 435.1291 [M+COO]^−^	1.2	C_20_H_21_O_8_	227.0713(100)	228(16), 225(9), 185(2)	[19]
22	Procyanidin dimer monogallate	4.1	225, 280	729.1446	2.1	C_37_H_29_O_16_	407.0777(100)	289(71), 577(47), 441(31), 451(27)	[3]
23	Unknown	5.5	220, 275	269.0147	1.3	C_7_H_9_O_11_	189.0584 (100)		-
24	Piceid	6.0	220, 304, 315	389.1245	−0.8	C_20_H_21_O_8_	227.0716(100)	228(13), 185(2), 225(0.5)	[19]
25	Epicatechin-3-O-gallate	6.3	220, 279	441.0825	0.6	C_22_H_17_O_10_	169.0145(100)	289(51), 125(22), 245(14)	[19]
26	Procyanidin trimer monogallate	6.7–6.8	225, 280	1017.2127	−3.1	C_52_H_41_O_22_	729.1431(100)	577(31), 865(30), 287(28), 441(19)	[3]
27	Procyanidin tetramer monogallate	7.3	225, 280	652.1307 [M−2H]^2−^, 1305.2673	−6.2	C_67_H_53_O_28_	125.0241(100)	169(94), 289(52), 407(25), 451(11), 577(7), 729(6)	[3]
28	Resveratrol hexoside	7.8	220, 304, 315	389.1242	−0,1	C_20_H_21_O_8_	227.0712(100)	228(11), 185(1), 143(0.5)	[19]
29	Resveratrol derivative	8.4	220, 282, 325	431.1356	−2.0	C_22_H_23_O_9_	227.0722(100)	228(14), 185(1)	[19]
30	Resveratrol hexoside	8.7	220, 304, 315	389.1249	−1.9	C_20_H_21_O_8_	227.0718(100)	228(15), 185(2), 143(0.5)	[19]
31	Unknown	11.2	220, 279, 307	253.0513	−2.5	C_15_H_9_O_4_	253.0513(100)	254 (17), 224(9), 209(7), 197(3), 135(3)	-
32	Torachrysone- hexoside	12.3	226, 266, 325sh	407.1359	−1.1	C_20_H_23_O_9_	245.0845(100)	246(14), 230(11)	[19]
33	Emodin-glucoside	12.8	221, 269, 281, 423	431.099	−1.5	C_21_H_19_O_10_	269.0465(100)	431(50),311(5)	[19]
34	Emodin-8-O-(6′-O-malonyl)-glucoside	14.2	220, 281, 423	517.0991	−0.7	C_24_H_21_O_13_	473.1098(100)	269(68), 311(3)	[19]
35	Sulfonyl torachrysone	14.8	220, 312	325.0395	−2.3	C_14_H_13_O_7_S	245.0829(100)	230 (34)	[6]
36	Emodin	25.4	221, 248, 267, 288, 430	269.0464	−3.1	C_15_H_9_O_5_	269.0464(100)	225(29), 241(10), 197(2), 181(1)	[19]
37	Physcion *	29.5	222, 266, 288, 430	285.0757 [M-H]^+^	−1.9	C_16_H_13_O_5_ [M-H]^+^	-		[19]

HMDB ID: The Human Metabolome Database, Physcion*: observed only in the positive mode.

**Table 4:** Content of analyzed compounds in studied decoction.

Analyte	*R. japonica* Decoction				
	(μg/mL of Liquid Decoction)	%CV	(mg/g of Dry Decoction)	%CV	(mg/g Dry Plant Material)
Piceid	22.60	0.23	4.52	0.23	0.83
Resveratrol	-	-	-	-	-
Vanicoside B	-	-	-	-	-
Vanicoside A	-	-	-	-	-
Emodin	1.41 *	0.40	0.28	0.40	0.05
Physcion	1.26 *	2.31	0.25	2.31	0.05

*—level below LOQ but above LOD.

## Data Availability

Data is contained within the article.
